# Advances in Longevity: The Intersection of Regenerative Medicine and Cosmetic Dermatology

**DOI:** 10.1111/jocd.70356

**Published:** 2025-07-17

**Authors:** Diala Haykal, Frederic Flament, Mukta Shadev, Pascale Mora, Cristina Puyat, Brigitte Dréno, Qian Zheng, Hugues Cartier, Michael Gold, Steven Cohen

**Affiliations:** ^1^ Centre Laser Palaiseau Palaiseau France; ^2^ L'Oréal Research and Innovation Clichy France; ^3^ Manipal Hospital Bangalore Bangalore India; ^4^ Asian Stem Cell Institute Inc. Metro Manila Philippines; ^5^ Head of Procedural Dermatology Subspecialty in Cosmetic Dermatology Rizal Medical Center Metro Manila Philippines; ^6^ Nantes Université, INSERM, CNRS, Immunology and New Concepts in ImmunoTherapy, INCIT Nantes France; ^7^ L'Oreal Research and Innovation Clark New Jersey USA; ^8^ Private Practice Centre Médical Saint Jean d'Arras Arras France; ^9^ Clinical Research Center Gold Skin Care Center Nashville Tennessee USA; ^10^ FACES+ Plastic Surgery, Skin and Laser Center, Division of Plastic Surgery University of California San Diego California USA

**Keywords:** cosmetic dermatology, cosmetic legislations, cosmetic surgery, dermatology, regenerative medicine, stem cells

## Abstract

**Background:**

Aging is increasingly recognized as a modifiable biological process influenced by genetic, environmental, and lifestyle factors. Recent advances in regenerative medicine and artificial intelligence (AI) have reshaped the field of cosmetic dermatology, shifting the focus from temporary aesthetic improvements to long‐term interventions aimed at preserving skin vitality and longevity.

**Aim:**

This narrative review aims to synthesize emerging knowledge from 2010 to 2025 on the integration of regenerative strategies, biological modulators, immunologic regulation, microbiome modulation, and AI‐driven personalization in the context of aesthetic longevity. The review also discusses translational potential and ethical considerations surrounding these advancements.

**Methods:**

A targeted literature search was conducted using PubMed and Scopus to identify peer‐reviewed articles from 2010 to 2025. Search terms included “skin aging,” “stem cells,” “mitochondrial dysfunction,” “epigenetic reprogramming,” “artificial intelligence in dermatology,” and “skin microbiome.” Selected studies focused on regenerative and longevity‐based interventions with clinical relevance or future applicability in cosmetic dermatology.

**Results:**

Key findings were categorized into six interconnected domains: regenerative medicine, mitochondrial function, epigenetic modulation, immunological balance, microbiome resilience, and AI‐driven innovation. These pillars demonstrate a paradigm shift toward biologically informed, personalized strategies that aim to restore and sustain skin health at the molecular level.

**Conclusion:**

Cosmetic dermatology is undergoing a transformation toward integrative, proactive care that combines regenerative medicine, AI, and personalized interventions. These approaches offer promising, evidence‐based solutions for enhancing both aesthetic outcomes and long‐term skin function, while also raising important ethical and regulatory considerations for clinical implementation.

## 1 Introduction

Longevity, once a futuristic ideal, has become a clinical reality at the intersection of regenerative medicine and cosmetic dermatology [1, 2, 3]. Aging is now increasingly regarded as a modifiable process, shaped by genetic predisposition, lifestyle factors, and molecular pathways. Over the past 15 years, advancements in stem cell therapy, mitochondrial research, epigenetic reprogramming, and artificial intelligence (AI) have redefined the dermatologic approach to skin aging [4, 5]. This evolution marks a transition from reactive, surface‐level aesthetic treatments to science‐driven strategies that restore skin integrity at a cellular level. Regenerative dermatology seeks not only to rejuvenate appearance but to sustain skin vitality through targeted interventions. AI further personalizes this approach by enabling predictive diagnostics and adaptive treatment planning based on genomic, biometric, and behavioral data [6]. Recent investigations into cellular senescence, mitochondrial dysfunction, immunological aging, and microbiome dysbiosis have revealed new therapeutic targets for skin renewal. As these insights converge, they are reshaping the future of aesthetic medicine toward a proactive model grounded in biological longevity [7, 8].

This narrative review synthesizes emerging literature, highlighting how regenerative strategies, biological modulators, immune and microbiome‐centered approaches, and AI‐driven personalization are reshaping the landscape of longevity‐focused dermatologic care.

## 2 Methods

This review was conducted in accordance with the PRISMA guidelines to improve methodological transparency and ensure reproducibility. A comprehensive search was performed using the PubMed and Scopus databases, covering literature published between January 2010 and March 2025. The search strategy was based on a combination of MeSH terms and free‐text keywords including “skin aging,” “regenerative dermatology,” “stem cells,” “exosomes,” “mitochondrial dysfunction,” “epigenetic reprogramming,” “artificial intelligence in dermatology,” “large language models,” and “skin microbiome.”

All articles retrieved were screened for relevance to regenerative and longevity‐based interventions applicable to dermatologic care. The inclusion criteria comprised peer‐reviewed studies focusing on human subjects or human‐derived tissue models, reporting on interventions with clinical or translational significance to aesthetic dermatology. Eligible study designs included clinical trials, observational studies, systematic reviews, meta‐analyses, and mechanistic investigations with potential applicability to clinical practice. Articles were excluded if they were non‐peer‐reviewed opinion pieces, animal‐only studies without translational insights, or if they lacked relevance to dermatologic or aesthetic applications.

**FIGURE 1:**
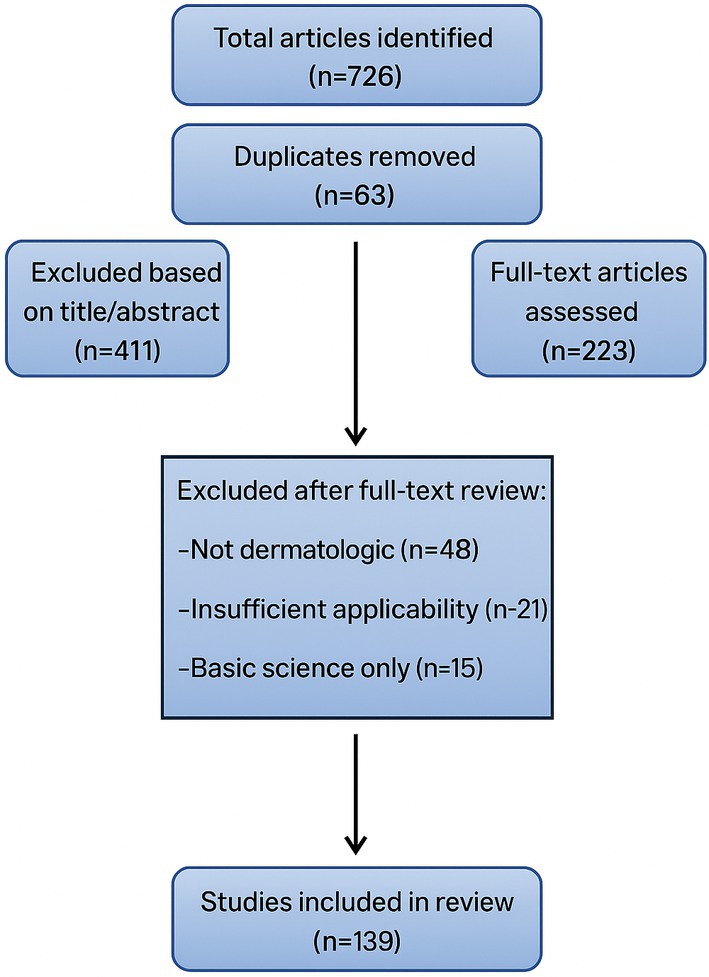
Flowchart.

The initial search yielded a total of 726 articles. After the removal of duplicates, 634 unique records were screened by two independent reviewers. Based on title and abstract evaluation, 411 articles were excluded. The remaining 223 full‐text articles were retrieved and assessed for eligibility. Following a detailed review, 139 studies were deemed suitable and included in the qualitative synthesis. Reasons for exclusion at the full‐text stage included lack of dermatologic relevance, insufficient clinical applicability, or focus on basic science without translational orientation (Figure 1).

## 3 Results

The landscape of aesthetic dermatology has been redefined by rapid advances in regenerative medicine and technological innovation. Rather than treating aging as an inevitable decline, emerging approaches aim to restore cellular function, reverse molecular damage, and extend skin health span. This review organizes the key findings into six interconnected pillars, each representing a critical domain influencing skin longevity: regenerative medicine, mitochondrial function, epigenetic modulation, immunological balance, microbiome resilience, and AI‐driven personalization. Together, these themes illustrate a shift from superficial corrections toward biologically informed, long‐term rejuvenation strategies (Table 1).

**TABLE 1:** Clinical readiness and evidence landscape of emerging technologies in cosmetic dermatology.

Technology/Approach	Maturity level	Evidence type	Notes
Platelet‐rich plasma (PRP)	Established	Multiple RCTs, Meta‐analyses	Clinically approved for skin rejuvenation
Adipose‐derived stem cell therapy	Established	Clinical studies, case series	Widely used in regenerative aesthetics
Exosomes (MSC‐derived)	Promising	Early clinical data, in vitro models	Increasing interest; not yet standardized
Nicotinamide riboside/NAD^+^ precursors	Promising	Phase I–II clinical trials	Oral and topical formulations being explored
Epigenetic reprogramming (yamanaka factors)	Experimental	Preclinical models, animal studies	Safety and efficacy in humans not yet established
AI‐guided diagnosis (LLMs, imaging tools)	Promising	Pilot studies, growing clinical deployment	Integration in practice emerging
Artificial mitochondrial transfer (AMT/T)	Experimental	In vitro and animal studies only	Conceptual; not ready for clinical use
Microbiome‐targeted topicals	Promising	Early‐stage trials	Formulations vary; need more robust RCTs
Wearables for skin health monitoring	Promising	Observational and pilot trials	Consumer and clinic crossover technologies emerging

**FIGURE 2:**
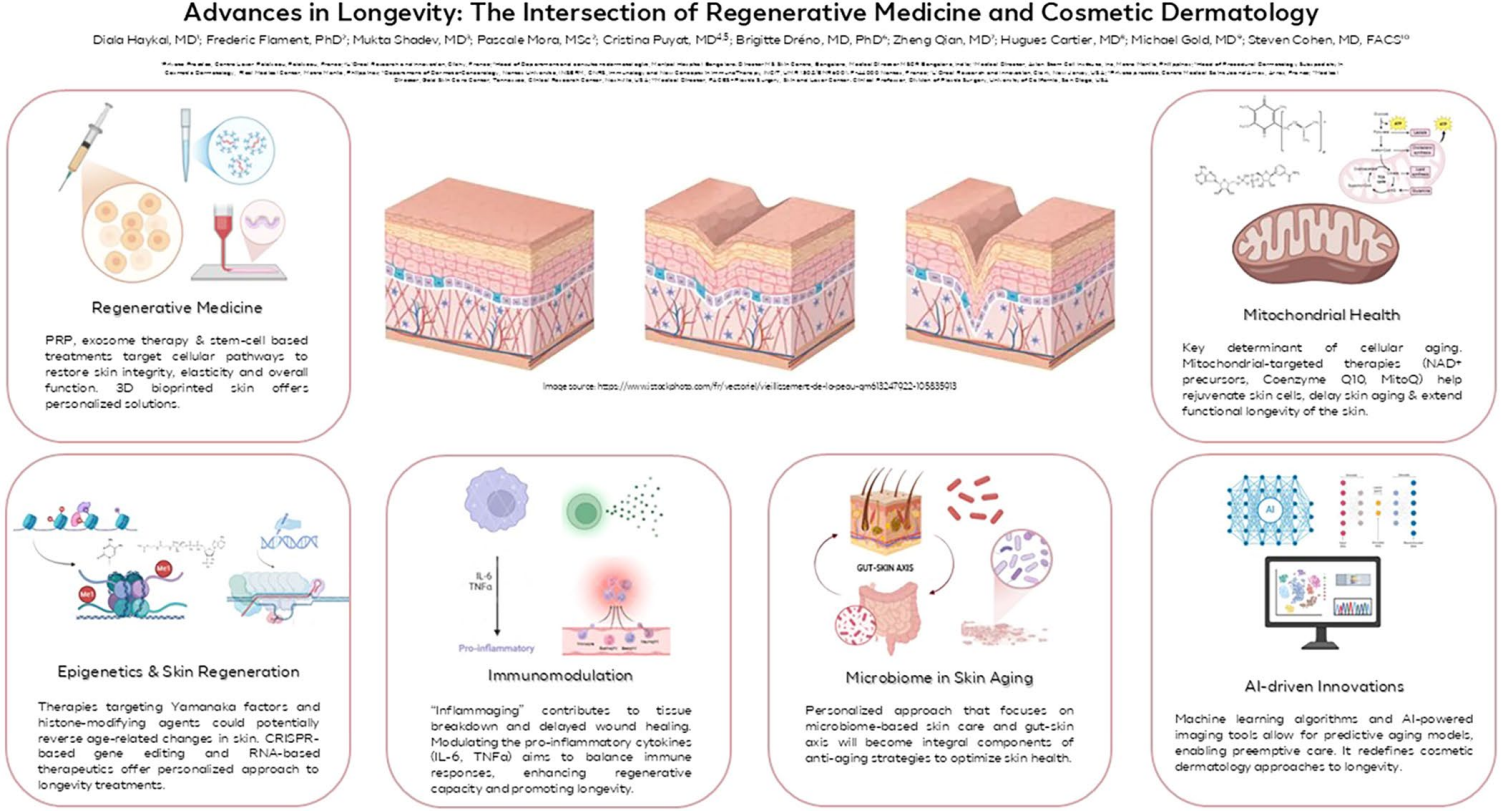
Key advancements at the intersection of regenerative medicine and cosmetic dermatology, highlighting six major pillars: Regenerative Medicine, Mitochondrial Health, Epigenetics and Skin Regeneration, Immunomodulation, Microbiome in Skin Aging, and AI‐driven Innovations.

The graphical abstract (Figure 2) provides a visual overview of key advancements at the intersection of regenerative medicine and cosmetic dermatology, highlighting six major pillars: Regenerative Medicine, Mitochondrial Health, Epigenetics & Skin Regeneration, Immunomodulation, Microbiome in Skin Aging, and AI‐driven Innovations.

### 3.1 Regenerative Medicine: A New Era of Rejuvenation

Regenerative medicine focuses on harnessing the body's natural healing mechanisms to repair and restore tissues [9]. Regeneration is a complex, multi‐step biological process that re‐establishes tissue architecture and function following damage or age‐related degeneration [10]. Unlike simple tissue repair, which may result in fibrosis or functional impairment, true regeneration involves coordinated cellular proliferation, differentiation, and immune response modulation to restore homeostasis [11]. In dermatology, regenerative medicine leverages these mechanisms to enhance collagen synthesis, reinforce skin barrier function, and counteract cellular senescence, offering long‐term therapeutic benefits beyond conventional aesthetic treatments [12]. In cosmetic dermatology, therapies such as platelet‐rich plasma (PRP), exosome therapy, and stem cell‐based treatments are at the forefront of this innovative field [13]. These innovations go beyond superficial improvements, targeting cellular pathways to restore skin integrity, elasticity, and overall function. Recent studies highlight the role of mesenchymal stem cells (MSCs) in tissue regeneration and longevity [14]. Human MSC‐derived exosomes have demonstrated the ability to enhance wound healing, reduce inflammation, and stimulate fibroblast proliferation, leading to improved skin rejuvenation outcomes [13]. Additionally, studies have shown that exosomes help regulate oxidative stress, a critical factor in the aging process, by delivering bioactive molecules that support cell viability and function [15, 16, 17, 18]. Stem cell therapy has gained attention for its ability to enhance collagen synthesis and tissue repair. Adipose‐derived stem cells (ADSCs) have demonstrated regenerative potential in skin rejuvenation through multiple mechanisms, including collagen synthesis enhancement, fibroblast proliferation, and reduction of oxidative stress. Recent studies highlight their role in improving skin elasticity, texture, and overall quality through exosome‐mediated signaling and paracrine effects [19, 20, 21, 22, 23]. Similarly, exosomes and growth factors facilitate intercellular communication, modulating inflammation and accelerating regeneration [24]. Research is also exploring induced pluripotent stem cells (iPSCs), which have the ability to transform into specialized cell types, offering potential applications in customized tissue regeneration [13, 25]. However, the use of iPSCs remains subject to strict ethical and regulatory constraints in certain countries, where their application in clinical practice is currently restricted. Another breakthrough in dermatological bioengineering is 3D bioprinting and tissue engineering, where the development of bioprinted skin grafts offers a future alternative to traditional fillers and skin transplants, providing personalized reconstructive and regenerative‐like solutions [26]. Efforts are focused on engineering bioactive scaffolds that mimic the extracellular matrix, creating a supportive environment for cell growth and collagen production [27]. This technology has the potential to revolutionize wound healing, scar management, and overall skin rejuvenation by providing structurally and functionally optimized tissue replacements [28, 29]. As regenerative medicine continues to evolve, its integration with longevity‐focused dermatology holds great promise in creating more effective, long‐term solutions for skin aging and rejuvenation [3, 30]. The future of cosmetic dermatology lies in utilizing regenerative therapies that not only improve aesthetics, but also restore the underlying biological health of the skin [31].

### 3.2 The Role of Mitochondrial Condition in Skin Longevity

Mitochondrial function is a key determinant of cellular aging, influencing energy production, oxidative stress, and skin regeneration. Recent research has shown that mitochondrial dysfunction contributes to the breakdown of collagen and elastin, leading to premature aging [32, 33, 34, 35]. Emerging therapies such as mitochondrial‐targeted antioxidants, NAD^+^ precursors, and autophagy‐stimulating compounds are being explored to rejuvenate skin cells at the mitochondrial level [36, 37, 38, 39, 40]. Mitochondrial‐targeted antioxidants, such as Coenzyme Q10 and MitoQ, help neutralize oxidative stress, and improve mitochondrial efficiency [41, 42, 43]. NAD^+^ precursors like nicotinamide riboside and nicotinamide mononucleotide have shown promise in restoring cellular energy metabolism and enhancing DNA repair mechanisms, thereby reducing the effects of aging on the skin [44, 45]. With continued advancements in mitochondrial‐targeted therapies, cosmetic dermatology is poised to adopt a more integrative approach to skin aging that enhances aesthetics and supports long‐term cellular health. Building upon these advancements, research conducted by Balcazar et al. proposes that artificial mitochondrial transfer/transplantation (AMT/T) could serve as a strategy to replenish the endogenous mitochondrial pool with beneficial mitochondria. However, this concept remains in the early stages of in vitro research, with significant challenges ahead before translation into clinical applications. Despite the remaining questions regarding intracellular interactions and transfer mechanisms, mitochondria play a crucial role in skin health. The integrity of mitochondria within a complex cellular network contributes to essential functions such as photoprotection, homeostasis, and overall skin longevity [46]. While promising, these therapies are still in experimental phases, and further studies are required to evaluate their feasibility, safety, and long‐term benefits for human applications [13, 46].

### 3.3 Epigenetics and Skin Regeneration

Epigenetics plays a crucial role in the aging process by regulating gene expression without altering DNA sequences. Factors such as environmental exposure, diet, and lifestyle choices influence epigenetic modifications, which can accelerate or decelerate aging [47, 48, 49]. Research in epigenetic reprogramming suggests that certain compounds, including Yamanaka factors and histone‐modifying agents, can reverse age‐related changes in skin cells [50, 51]. By targeting specific epigenetic pathways, scientists are exploring ways to reset cellular aging, paving the way for regenerative treatments that could sustain youthful skin for longer periods. Recent advances in epigenetic therapies have focused on DNA methylation patterns and histone acetylation, which influence how genes associated with aging are expressed [52, 53]. Additionally, CRISPR‐based gene editing is being explored as a potential means of directly modifying epigenetic markers to delay cellular aging [54, 55]. Another promising area of research involves the use of RNA‐based therapeutics, which can influence gene expression in a reversible manner. Scientists are investigating small interfering RNAs (siRNAs) and microRNAs (miRNAs) as potential tools to regulate genes responsible for collagen degradation and skin inflammation [56, 57, 58, 59]. This approach holds potential for precision dermatology, where specific epigenetic changes can be modulated to slow aging at a cellular level. A fundamental question remains: will these epigenetic modifications 1 day become transmissible? If epigenetic interventions can be inherited across generations, they could revolutionize regenerative medicine by providing long‐term benefits beyond a single individual. However, this also raises ethical considerations regarding genetic interventions and unintended consequences. Future research will need to explore whether these changes can be safely and predictably passed on or whether epigenetic therapies will remain transient, requiring periodic intervention for sustained effects [60].

### 3.4 Immunomodulation and Skin Longevity

The skin's immune system plays a significant role in aging and tissue repair. Chronic low‐grade inflammation, often referred to as “inflammaging,” contributes to tissue breakdown and delayed wound healing [61, 62, 63, 64, 65]. This persistent inflammation state disrupts the skin barrier, accelerates collagen degradation, and impairs the skin's ability to repair itself efficiently. As a result, addressing inflammaging has become a focal point in longevity‐based dermatological research. Targeted immunomodulatory treatments, including cytokine‐targeting biologics, mesenchymal stem cell‐derived factors, and exosome therapies, are being investigated for their ability to regulate inflammation while promoting skin repair [66, 67]. By modulating the activity of pro‐inflammatory cytokines such as interleukin‐6 (IL‐6) and tumor necrosis factor‐alpha (TNF‐α), these therapies aim to reduce chronic inflammation and restore homeostasis in the skin microenvironment [68]. The microbiome plays a crucial role in immune regulation, and while some research suggests that probiotics and prebiotics may influence immune responses, their clinical efficacy in dermatology remains under investigation [69]. Current evidence indicates that maintaining a balanced skin microbiome contributes to skin resilience and reduced inflammation, but the effectiveness of probiotic and prebiotic‐based cosmetic formulations is still debated [70, 71]. As research progresses, immunomodulatory interventions are being explored for their role in personalized anti‐aging regimens, with the goal of preventing age‐related skin deterioration and optimizing the skin's natural healing potential [63, 72].

### 3.5 The Influence of Microbiome in Skin Aging

The skin microbiome is a crucial factor in maintaining skin health, barrier function, and overall longevity. Research has demonstrated that an imbalanced microbiome can accelerate aging by increasing susceptibility to inflammation and oxidative stress [73]. The diversity and stability of microbial populations play a key role in preserving skin resilience, hydration, and immune responses [74, 75, 76]. Probiotic‐based skincare, microbiome‐friendly formulations, and postbiotic treatments are being explored as potential strategies to support microbial diversity and improve skin resilience. However, current studies in this field often lack rigorous clinical validation, and their efficacy remains to be conclusively demonstrated [71, 77]. Some research suggests that specific bacterial strains, such as Lactobacillus and Bifidobacterium, contribute to skin barrier function by enhancing natural defenses, reducing transepidermal water loss, and mitigating signs of aging, but further well‐designed clinical trials are needed to validate these findings [78, 79].

Personalized microbiome‐based skincare and precision probiotics will soon become integral components of anti‐aging strategies, leveraging an individual's unique microbiota composition to optimize skin health. Future directions in microbiome research aim to further elucidate how microbial diversity shifts with age and how targeted interventions can restore balance [73, 80]. With continued advances in this field, microbiome modulation would become a key pillar of longevity‐focused dermatology, offering non‐invasive yet highly effective means to preserve youthful and resilient skin [81, 82, 83].

### 3.6 Lifestyle Interventions and Longevity Optimization

Beyond medical treatments, lifestyle modifications have been shown to significantly influence longevity and regenerative capacity [84, 85, 86]. Intermittent fasting, caloric restriction, and plant‐based diets rich in polyphenols have been linked to enhanced cellular repair mechanisms and reduced signs of aging [87, 88, 89, 90]. Regular physical activity induces the release of myokines, signaling proteins that enhance collagen synthesis and skin elasticity [91, 92]. Furthermore, sleep optimization is crucial for skin barrier recovery, as deep sleep facilitates growth hormone secretion and reduces cortisol‐driven inflammation [93, 94, 95, 96, 97]. Advancements in skin longevity diagnostics are now integrating (Online + Offline) approaches, combining in‐clinic assessments with real‐time, data‐driven insights to optimize skincare and medical treatments [98]. Cutting‐edge diagnostic tools in clinics and retail spaces enable deep skin analysis, identifying subclinical disorders before they become visible. These assessments can be enhanced through biomarker profiling via blood, urine, or saliva analysis, offering insights into inflammation levels, metabolic health, and oxidative stress markers [99, 100, 101]. Furthermore, next‐generation health monitoring extends beyond clinical settings. Wearable devices, AI‐powered scoring systems, and real‐time sensors now allow continuous tracking of skin hydration, UV exposure, and overall physiological status [33, 102, 103, 104, 105, 106, 107]. Integrating these non‐invasive strategies with regenerative therapies provides a holistic approach to optimizing aesthetic longevity. The synergy between lifestyle choices and cutting‐edge medical advancements will continue to shape the future of aging, offering individuals sustainable and effective solutions for maintaining youthful and resilient skin [108].

### 3.7 AI‐Driven Innovations in Longevity and Cosmetic Dermatology

Artificial intelligence (AI) is transforming longevity‐focused dermatology by enabling precision diagnostics and highly personalized treatment plans [7, 8]. Machine learning algorithms analyze large datasets that combine genetic predispositions, environmental exposures, lifestyle factors, and real‐time patient data. This integration allows for accurate prediction of skin aging trajectories and the development of tailored, proactive interventions [109, 110].

Advanced AI‐powered imaging tools, such as deep‐learning skin analysis platforms and multispectral imaging systems, can detect early molecular signs of aging, sun damage, and pigmentation abnormalities at a microscopic level [111, 112, 113]. These technologies help clinicians identify subclinical skin changes before visible aging signs appear, enabling early and targeted treatments. In addition, computer vision and 3D skin mapping technologies offer detailed assessments of skin texture, hydration, and elasticity, creating a comprehensive longevity‐focused dermatologic profile [114, 115].

Predictive aging models now allow simulation of future skin aging based on a patient's genetic and environmental profile, facilitating preemptive skincare strategies. AI‐assisted epigenetic tracking represents another breakthrough, enabling the monitoring of biological aging markers and dynamic adjustment of interventions based on real‐time treatment response [116]. This paves the way for adaptive anti‐aging protocols that evolve alongside the patient's biology.

In procedural dermatology, AI is enhancing both precision and safety. Robotic‐assisted systems and AI‐guided injectors minimize human error and ensure optimal injection depth and distribution, yielding more natural and durable aesthetic outcomes [7, 8]. AI‐powered laser platforms can automatically calibrate settings in real time based on skin analysis, improving results in treatments such as fractional resurfacing, pigmentation correction, and collagen remodeling [117]. The integration of AI with regenerative medicine is driving the emergence of fully customized anti‐aging regimens. These protocols incorporate real‐time biological monitoring, automated treatment adjustments, and long‐term predictive analytics, shifting the dermatologic paradigm from reactive care to proactive, personalized longevity medicine [118, 119, 120, 121].

Beyond imaging and predictive modeling, large language models (LLMs) are becoming key components of AI integration in dermatology. These tools support clinical decision‐making by analyzing and generating medical text, offering diagnostic assistance, treatment planning, and patient education based on unstructured clinical data. LLMs can synthesize patient history, describe imaging inputs, and suggest therapeutic pathways, enhancing both accuracy and efficiency in dermatologic care [122, 123, 124]. Their application in common conditions such as acne and rosacea further demonstrates their practical relevance [124]. When integrated into multimodal systems alongside computer vision and biosensor data, LLMs have the potential to transform patient–physician communication, personalize interventions, and provide real‐time procedural support during aesthetic treatments.

However, several limitations must be acknowledged. Many AI models rely on datasets that may lack diversity, potentially leading to biased outcomes and reduced accuracy in underrepresented populations. The use of predictive algorithms in clinical settings also raises questions about over‐reliance on technology without sufficient clinical validation. Moreover, while AI can offer dynamic treatment plans, these models require continuous data input and rigorous regulatory oversight to ensure safety and efficacy. Concerns around data privacy, algorithmic transparency, and equitable access remain significant and must be addressed through ethical guidelines, robust governance, and inclusive AI training datasets [125, 126]. As AI continues to advance, ensuring transparency, ethical implementation, and broad accessibility will be essential for maintaining patient trust and maximizing the technology's potential in cosmetic dermatology and longevity care.

### 3.8 The Future of Cosmetic Dermatology and Longevity

The convergence of regenerative medicine and AI is revolutionizing cosmetic dermatology. AI‐driven epigenetic reprogramming could help decode and reset aging markers at a molecular level. Breakthroughs in longevity peptides and mRNA‐based therapies are paving the way for regenerative treatments [127]. Small Peptides such as GHK‐Cu (Gly‐His‐Lys‐Cu) have shown strong potential in stimulating skin regeneration, reducing oxidative stress, and enhancing extracellular matrix remodeling [78, 128, 129]. Meanwhile, mRNA technology, widely recognized for its role in vaccines, is now being explored for applications in skin rejuvenation and cellular reprogramming [130, 131].

Looking ahead, the field would explore advancements in bioengineered skin grafts incorporating stem cells, AI‐powered predictive analytics for longevity optimization, and genetic‐based skincare tailored to an individual's unique DNA profile [132]. Additionally, wearable biotechnology will play a significant role in continuously monitoring skin health, enabling real‐time adjustments to treatment plans [105]. The integration of AI‐driven robotics for non‐invasive procedures and tissue engineering could further redefine anti‐aging strategies, offering more precise and personalized interventions. Future personalized longevity clinics will integrate AI diagnostics, regenerative therapies, and tailored anti‐aging solutions [133]. This shift from reactive to proactive aging management ensures patients receive scientifically grounded, individualized treatments, optimizing skin health, and longevity for the long term.

### 3.9 Ethical and Regulatory Considerations

As the landscape of cosmetic dermatology shifts toward longevity‐focused interventions, ethical and regulatory considerations must evolve in tandem [134]. The use of AI‐driven diagnostics, gene editing, and regenerative therapies raises questions about data privacy, algorithmic bias, accessibility, and long‐term safety [135, 136].

Transparency and oversight are essential. AI‐assisted diagnostics and treatment planning must be rigorously validated to avoid over‐reliance on unverified models. Regulatory bodies should mandate clear standards for algorithm performance, explainability, and clinical accountability, especially in aesthetic contexts where subjective outcomes can compound ethical ambiguity [125, 137, 138, 139]. The expansion of genetic and regenerative interventions also demands caution. Therapies involving exosomes, iPSCs, or epigenetic reprogramming must adhere to strict protocols for patient consent, risk disclosure, and post‐intervention monitoring. As these approaches move closer to clinical application, the potential for off‐target effects and unforeseen consequences necessitates robust regulatory review.

Equity remains a pressing concern. Many of the technologies described, such as exosome‐based therapies, epigenetic reprogramming, and AI‐guided diagnostics, are likely to be expensive and initially accessible only to affluent populations, potentially deepening existing disparities in access to aesthetic and preventive care. Without proactive efforts to democratize access through public health integration, ethical pricing strategies, and inclusive trial design, longevity dermatology risks reinforcing socioeconomic stratification in appearance‐related healthcare. Equally important are the psychosocial consequences of extending the “youthful” aesthetic through biotechnological means. These interventions may shift societal perceptions of aging, normalize continuous cosmetic optimization, and create psychological pressures around beauty, aging, and self‐worth, particularly among younger individuals. Addressing these impacts requires not only informed consent but also a broader societal dialogue around identity, aging, and the ethical boundaries of appearance medicine.

Moving forward, future longevity clinics and personalized anti‐aging programs should operate within clear ethical and medical guidelines to balance innovation with patient safety, equity, and informed choice. Through collaboration between researchers, ethicists, and regulatory bodies, we can harness the full potential of these advancements while maintaining ethical integrity and public trust [8].

### 3.10 Challenges in Clinical Translation and Global Adoption

While scientific innovation is rapidly advancing in cosmetic and longevity dermatology, several challenges impede seamless clinical translation. Artificial intelligence tools, though promising, are often developed on narrow or homogeneous datasets, resulting in algorithmic bias and limited generalizability. Without active correction and inclusive data sourcing, these tools risk exacerbating disparities in diagnostic and treatment outcomes. Furthermore, over‐reliance on AI predictions, especially without sufficient clinical validation, may compromise clinical judgment and patient safety.

Compounding these concerns is the global heterogeneity in regulatory oversight. Stem cell therapies, gene editing, and AI‐assisted diagnostics are subject to vastly different frameworks across countries, ranging from permissive innovation environments to strict prohibitions. This regulatory fragmentation hinders international collaboration and consistent implementation of longevity‐focused interventions. Harmonization efforts, including shared clinical evidence standards, interoperable validation protocols, and ethical oversight mechanisms, are essential to balance innovation with safety and equity on a global scale.

Similar concerns apply to emerging microbiome‐based interventions. While many topical probiotics and postbiotics are commercially available, few meet rigorous evidence thresholds. Clinicians should require support from randomized controlled trials with dermatologic endpoints, validated strain‐specific mechanisms, and reproducible human data before incorporating such interventions into routine practice. Until then, these therapies should be considered exploratory and primarily adjunctive. Beyond single‐modality approaches, the next frontier in longevity dermatology lies in combining diverse data streams into unified, personalized treatment strategies.

While the promise of personalized longevity care is compelling, its clinical implementation poses substantial challenges. Integrating complex layers of biological data, including genomic, epigenetic, microbiome, and lifestyle inputs, into everyday dermatologic decision‐making requires robust infrastructure, interoperability between diagnostic platforms, and advanced clinical decision support tools. Most dermatologists are not yet trained to interpret or act upon multi‐omics datasets, highlighting a critical need for multidisciplinary education and updated clinical guidelines. Moreover, achieving true personalization will require seamless collaboration between clinicians, AI systems, bioinformaticians, and patients themselves, alongside investment in secure data management systems. Until such capabilities are widely adopted, personalized anti‐aging protocols may remain limited to research settings or highly specialized clinics.

## 4 Discussion

The integration of regenerative and longevity‐focused interventions into dermatologic practice marks a significant shift from reactive to preventive and rejuvenative care. However, the clinical evidence supporting these technologies is heterogeneous, ranging from well‐established therapeutic modalities to innovations that remain primarily theoretical or in preclinical development. To provide clarity, we stratified the emerging technologies discussed in this review into three categories: established therapies with robust clinical evidence, promising interventions currently under early clinical investigation, and experimental approaches grounded in preclinical or conceptual research.

Established interventions such as PRP, autologous fat grafting, and ADSC therapies have amassed substantial evidence through randomized controlled trials and clinical implementation, underscoring their relevance and reproducibility in aesthetic dermatology. These therapies are now integrated into standard protocols for skin rejuvenation, volume restoration, and scar management.

In contrast, a number of promising technologies, including exosome‐based solutions, nicotinamide riboside and NAD^+^ precursors, and microbiome‐targeted topicals, are supported by preliminary human trials and translational research. While their mechanistic rationale is compelling, broader clinical validation remains necessary to define their long‐term efficacy and safety profiles. These interventions are actively being explored in early‐phase clinical trials and represent the forefront of innovation in skin regeneration.

At the frontier of dermatologic science are interventions still in their experimental phase. These include artificial mitochondrial transfer, Yamanaka factor‐induced partial reprogramming, and the application of AI and LLMs for predictive diagnostics and treatment personalization. While these approaches offer transformative potential, they currently rest on preclinical foundations or limited pilot data, and their translation into dermatologic practice will require rigorous clinical scrutiny and ethical consideration.

## 5 Conclusion

Cosmetic dermatology is increasingly shaped by regenerative medicine, AI, and system‐level approaches that target the biological foundations of skin aging. These innovations are shifting the field from aesthetic correction toward personalized restoration and longevity. Emerging strategies, ranging from stem cell‐based therapies and exosomes to epigenetic modulation, AI‐driven diagnostics, and microbiome‐targeted interventions, offer the promise of enhancing both appearance and long‐term skin health. However, the pace of innovation often exceeds clinical translation. Many of these technologies remain in early‐stage development, with timelines for implementation dependent on rigorous validation, safety profiling, and regulatory convergence. To maximize patient benefit, research investments should prioritize scalable interventions with early clinical promise and potential for integration into standard practice. Ethical considerations, including access, affordability, and social impact, must guide development to prevent widening disparities in dermatologic care.

Clinicians must navigate this evolving landscape by combining scientific curiosity with disciplined adherence to evidence‐based standards. Staying current with translational research, engaging in multidisciplinary collaboration, and maintaining a clear focus on patient‐centered outcomes are essential. While the focus on optimization and longevity grows, the foundational mission of dermatology, to treat conditions that cause suffering, must remain central. The integration of regenerative science, AI, and personalized medicine calls for a redefinition of dermatologic training. Future practitioners will require new skills in systems biology, digital health, and data interpretation. Updating medical education to reflect these needs will be crucial to preparing dermatologists to lead this next chapter of skin health and aesthetic medicine.

## Ethics Statement

The authors have nothing to report.

## Conflicts of Interest

The authors declare no conflicts of interest.

## Data Availability

The data that support the findings of this study are available on the references' part.
